# Analgesic Efficacy of Magnesium in Lower Third Molar Surgery: A Systematic Review and Meta-Analysis

**DOI:** 10.7759/cureus.95149

**Published:** 2025-10-22

**Authors:** Abdulaziz Owayed, Hanan A Alshammari, Mohammed Aljumaiaan, Dhari M Alshammari, Saad Almutairi, Yousef Al Dreess, Khaled Alansary, Yaqoub H Maarafi, Mariam H Alshammari, Fahad Alfadhli, Meshari Alanezi, Mohammad Almutairi

**Affiliations:** 1 Department of Dentistry, Ministry of Health, Kuwait City, KWT; 2 Department of Dentistry, Salwa Specialized Center Polyclinic, Ministry of Health, Kuwait City, KWT; 3 Department of Dentistry, Dasman Polyclinic, Ministry of Health, Kuwait City, KWT; 4 Department of Dentistry, Jaber Al-Ali Polyclinic, Ministry of Health, Kuwait City, KWT; 5 Department of Dentistry, Abdullah Abdulhadi Specialized Center Yarmouk Polyclinic, Ministry of Health, Kuwait City, KWT; 6 Department of Dentistry, Taima Dental Center Polyclinic, Ministry of Health, Kuwait City, KWT; 7 Department of Dentistry, Al-Osaimi Health Center Polyclinic, Ministry of Health, Kuwait City, KWT; 8 Department of Dentistry, Sulaibiya Polyclinic, Jahra, KWT

**Keywords:** ianb, inferior alveolar nerve block, lower third molar, magnesium, meta-analysis, mg, sip

## Abstract

Achieving an optimal analgesic effect in patients undergoing lower third molar surgery from the administration of an inferior alveolar nerve block (IANB) remains unclear. Magnesium, a N-methyl-D-aspartate receptor antagonist, has shown positive results in reducing pain following surgery. This systematic review and meta-analysis aimed to study whether the use of magnesium is effective in lower third molar surgery. We conducted a comprehensive search on PubMed, Web of Science, Scopus, and the Cochrane Library from inception to September 2023 for randomized controlled trials (RCTs) assessing the use of magnesium as an oral tablet or as an adjuvant to IANB in patients undergoing lower third molar surgery. The primary outcome was the pain scores following surgery at 24 hours, 48 hours, and 72 hours. Secondary outcomes included the number of analgesics consumed and the frequency of patients receiving supplemental analgesia. We calculated the standardized mean difference (SMD) and risk ratio along with their 95% confidence intervals (CIs) using a random-effects model. All analyses were performed using STATA 19MP. We included five RCTs with 299 patients in the analysis. Oral magnesium was associated with lower pain scores at 24 hours, 48 hours, and 72 hours following the surgery, with the following values, respectively: SMD = -0.7, 95% CI = -1.03 to -0.37, p < 0.001; I² = 0%; -0.59, 95% CI = -0.92 to -0.26, p < 0.001; I² = 0%; and -0.68, 95% CI = -1.01 to -0.35, p < 0.001; I² = 0% compared to the control group, with no significant difference between the adjuvant magnesium and the control group. The oral use of magnesium resulted in a significant reduction in pain scores at 24 hours, 48 hours, and 72 hours post-procedure compared to the control group. Further long-term RCTs with standardized protocols are recommended to confirm the current findings.

## Introduction and background

The surgical extraction of the impacted mandibular third molar (MTM) is one of the most common procedures in oral and maxillofacial surgery. The procedure involves mucoperiosteal flap elevation, bone removal, tooth separation, and flap suturing that contributes to pain, anxiety, and patient discomfort during and after the procedure [1]. Conventional pain management in MTM surgeries is the inferior alveolar nerve block (IANB), which blocks the sensory nerves from transmitting pain signals from the surgical sites to the brain [2]. Although being the gold standard, the IANB success rate is variable, and it fails frequently even when performed by skilled clinicians. Thus, pain relief protocols during MTM surgeries remain a significant challenge to clinicians.

Inflammation or a pre-existing infection at the site of the surgery can influence the effectiveness of anesthesia. Furthermore, inflammation induces tissue acidosis, which leads to trapping the anesthesia agent, along with increased blood flow, which together reduce the concentration of the anesthesia agent [3]. Previous studies have investigated different solutions to increase the IANB success rate, including magnesium as an adjuvant to the IANB solution [4]. The anti-inflammatory effect of magnesium and its analgesic potency have been studied extensively in surgical and postsurgical pain management protocols. As an important intracellular cation participating in numerous physiological functions, magnesium is a natural antagonist to the N-methyl D-aspartate (NMDA) receptor that contributes to a process called central sensitization and the wind-up phenomena, a state in which the neurons are hyperexcitable [5]. By blocking the NMDA receptor’s ion channels, it acts as a physiological calcium channel blocker, thus inhibiting the influx of calcium into the neuron, and preventing central sensitization, resulting in lower conductivity of pain signals [6]. In addition to these favorable effects on pain, it is also cost-effective and readily available, making it a strong candidate for local anesthesia as an adjuvant to IANB in oral and maxillofacial surgeries, including MTM extraction [7].

The aim of this systematic review and meta-analysis of randomized controlled trials (RCTs) is to critically appraise and synthesize the published evidence to determine the efficacy and safety of magnesium as an adjuvant to IANB in MTM extraction surgery.

## Review

Methodology

We conducted this systematic review and meta-analysis in adherence to the Preferred Reporting Items for Systematic Reviews and Meta-Analysis (PRISMA) [8] and the Cochrane Handbook of Systematic Reviews and Meta-analysis of Interventions guidelines [9].

Literature Search and Screening

A comprehensive electronic search was conducted on PubMed, Scopus, Web of Science (WOS), and Cochrane Library from inception to September 2025 for all relevant RCTs using the following search terms: (“Third Molar” OR “Third Molars” OR “Wisdom Tooth” OR “Wisdom Teeth”) AND (Surgery OR Extraction OR Removal OR Disimpaction) AND (Magnesium OR “Magnesium Sulfate” OR “Magnesium Sulphate” OR “Magnesium Citrate”). The detailed search strategy is presented in Table 1. Moreover, we conducted a comprehensive manual search for all references from the potentially included studies to ensure that all relevant studies were considered. The screening process was performed using a two-step approach. First, we screened titles and abstracts to identify all eligible studies, followed by a full-text screening to include only studies that met our eligibility criteria.

**Table 1:** Detailed search strategy for each database.

Database	Search terms	Search field	Search results
PubMed	(“Third Molar” OR “Third Molars” OR “Wisdom Tooth” OR “Wisdom Teeth”) AND (Surgery OR Extraction OR Removal OR Disimpaction) AND (Magnesium OR “Magnesium Sulfate” OR “Magnesium Sulphate” OR “Magnesium Citrate”)	All fields, English	12
Cochrane Library	(“Third Molar” OR “Third Molars” OR “Wisdom Tooth” OR “Wisdom Teeth”) AND (Surgery OR Extraction OR Removal OR Disimpaction) AND (Magnesium OR “Magnesium Sulfate” OR “Magnesium Sulphate” OR “Magnesium Citrate”)	All fields, English	17
Web of Science	(“Third Molar” OR “Third Molars” OR “Wisdom Tooth” OR “Wisdom Teeth”) AND (Surgery OR Extraction OR Removal OR Disimpaction) AND (Magnesium OR “Magnesium Sulfate” OR “Magnesium Sulphate” OR “Magnesium Citrate”)	All fields, English	3
Scopus	(“Third Molar” OR “Third Molars” OR “Wisdom Tooth” OR “Wisdom Teeth”) AND (Surgery OR Extraction OR Removal OR Disimpaction) AND (Magnesium OR “Magnesium Sulfate” OR “Magnesium Sulphate” OR “Magnesium Citrate”)	Titles, abstracts, keywords, English	20

Eligibility Criteria and Outcomes

We considered all eligible studies that met the following pre-specified criteria: (1) patients undergoing lower third molar surgery as our patients of interest; (2) the intervention was the use of magnesium either in an oral form or as an adjuvant to IANB; (3) the control group was IANB alone; and (4) RCTs. Additionally, we excluded studies that did not use magnesium as their intervention of choice, or studies with other lower molars, conference abstracts, or unpublished studies.

The primary outcome of interest was pain scores following the procedure, assessed using the Heft-Parker visual analog scale (HP-VAS) [10] and numerical VAS scale [11]. The HP-VAS assessment tool rated each patient using a 170-mm marked line divided into four categories with various terms describing the level of pain. The four categories were no pain (0 mm), mild pain (1 to 54 mm), moderate pain (55 to 113 mm), and severe pain (114 to 170 mm) [12]. Moreover, the numeric VAS tool was used to categorize pain on a 10-point rating scale, where “0” indicated no pain and “10” indicated the worst pain. Moreover, the secondary endpoints were the mean number of consumed analgesics and the frequency of patients who received supplemental analgesia.

Quality Assessment

The risk domains of the included studies were assessed using the risk of bias assessment tool for RCTs version 2, proposed by the Cochrane risk of bias tool for randomized clinical trials (ROB-2) [13]. Version 2 of the tool assesses five domains, i.e., bias from the randomization process, deviations from the intended interventions, missing outcome data, measurement of the reported outcomes, and selection of the reported outcomes. Each risk domain was categorized as low risk, some concerns, and high risk, and an overall risk of bias. Any further discrepancies were resolved by discussion.

Data Extraction and Statistical Analysis

A standardized Excel sheet was used to extract the required data from the included RCTs and patients as follows: (1) baseline characteristics of the included patients, including age, gender, and baseline pain score; (2) a summary of the included RCTs, including study design, sample size, pain assessment scale, and IANB technique; (3) risk of bias domains; and (4) the reported outcomes of interest.

Continuous data were extracted as mean change from the baseline to values following the procedure, standard deviations (SDs), and the total number of included patients. The reported data were then pooled as a standardized mean difference (SMD) with 95% confidence intervals (CIs) using the Der-Simonian-Laird random-effect model [9]. Additionally, dichotomous data were extracted and pooled as a risk ratio (RR) with its 95% CI using the above-declared random-effects model. Heterogeneity was assessed using the Cochrane Q test, and the I² measure was determined across all studies. A p-value less than 0.05 and I² ≥50% were considered as significant heterogeneity among the included studies. The packages “meta esize” and “meta forest plot” were used on STATA 19MP software to pool the effect estimates and the corresponding 95% CIs.

Results

**Figure 1:**
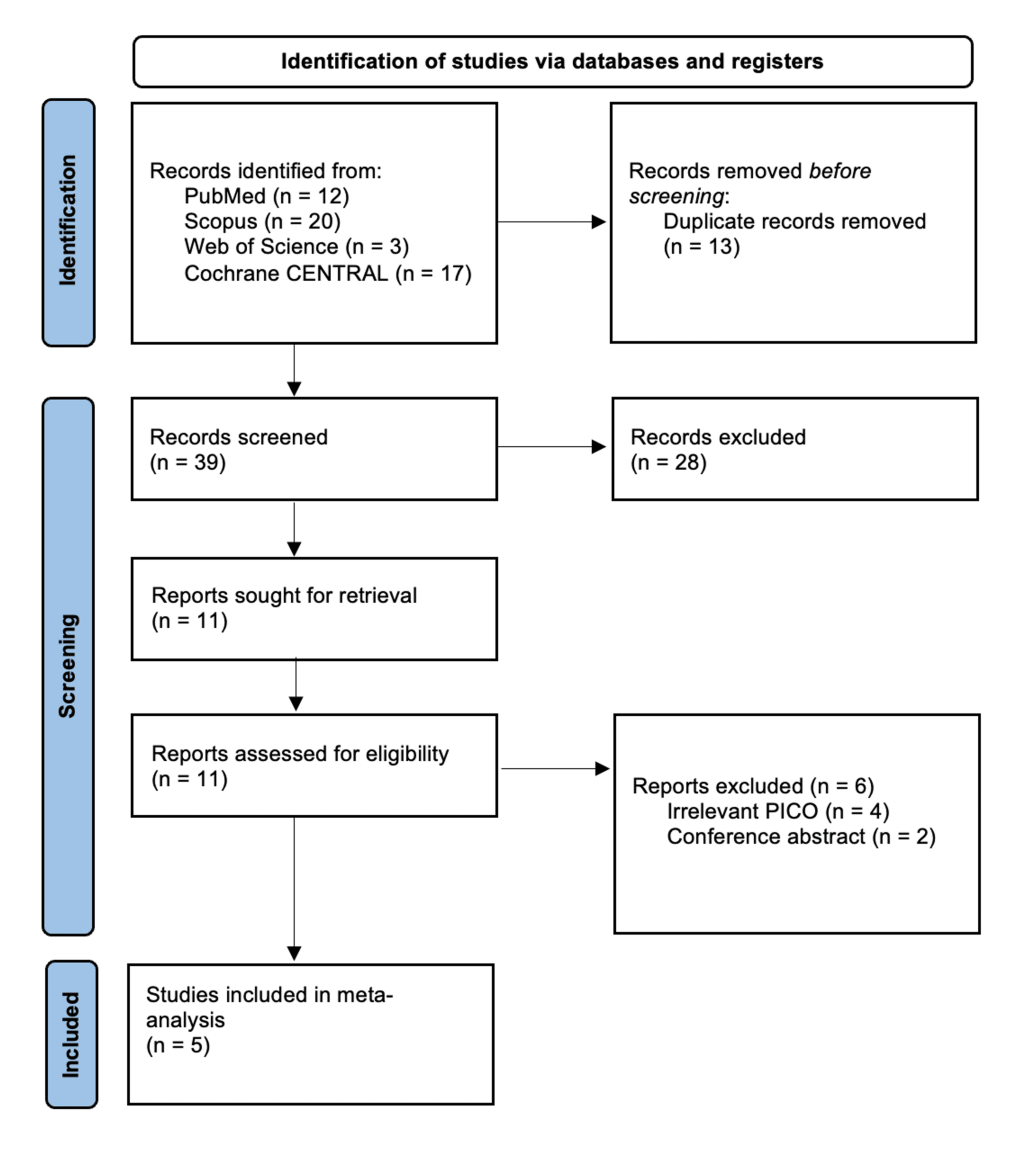
Preferred Reporting Items for Systematic Reviews and Meta-Analysis (PRISMA) flow diagram outlining the study selection process.

Our search of the literature yielded 52 citations, of which 41 were excluded following title and abstract screening and removal of duplicates. A total of 11 articles were eligible for full-text screening, of which five RCTs were finally included in this systematic review and meta-analysis [14-18]. The selection process of the citations is shown in the PRISMA flowchart (Figure 1).

Study Characteristics and Quality Assessment

The five included RCTs comprised 299 patients who underwent surgical removal of the lower third molars. Overall, 175 (58.5%) patients received magnesium, and 124 (41.5%) patients received IANB alone. The mean age of the included patients was 23.56 years, with a total of 141 (47.2.8%) patients being females. Detailed baseline characteristics of the included patients are presented in Table 2. The included RCTs were reported from India, Thailand, and Croatia, were published between 2017 and 2025, and included patients requiring bilateral or single MTM extraction. A summary of the included studies is presented in Table 3.

**Table 2:** Baseline variables of the included patients. NA: not available

Study ID	Arm	Number of patients	Age (years)		Gender (n, %)		Mouth opening	Baseline pain (mean, SD)
			Mean	SD	Female	Male		
Singh et al. (2025) [17]	Magnesium	26	23.3	3.78	28 (53.8)	24 (46.2)	NA	NA
	Control	26						
Jerkovic et al. (2020) [14]	Lozenge magnesium	20	24.25	3.26	21 (27.5)	19 (23.8)	52.85 ± 8.31	2.33 ± 2.84
	Lozenge control	20					53.93 ± 6.47	2.78 ± 3.24
	Tab magnesium	20	24.15	3.58	20 (25)	20 (26.2)	51.05 ± 5.77	3.36 ± 3.42
	Tab control	20					50.95 ± 5.85	2.79 ± 2.71
Powcharoen et al. (2025) [16]	Magnesium 150 mg	25	20.8	2.32	19 (76)	6 (24)	NA	NA
	Magnesium 250 mg	24	20.83	2.77	9 (37.5)	15 (62.5)	NA	
	Control	23	21.34	3.4	13 (56.5)	10 (43.5)	NA	
Nimkulrat et al. (2025) [15]	Magnesium	26	20.07	1.41	12 (46.15)	14 (53.85)	NA	16.04 ± 8.80
	Control	24	21.25	2.25	14 (58.33)	10 (41.67)		
Tejashree et al. (2021) [18]	Magnesium	35	29.2	8.6	15 (42.9)	20 (57.1)	NA	NA
	Control	35	27.4	10.8	17 (48.6)	18 (51.4)	NA	

**Table 3:** Summary of the included studies. MTMs: mandibular third molars; NA: not available; RCT: randomized controlled trial; HP-VAS: Heft-Parker visual analog scale; VAS: visual analog scale; ASA: American Society of Anesthesiologists

Study ID	Study design	Time of the study	Patient characteristics	Inclusion criteria	Exclusion criteria	Intervention	Control	Intervention form	Pain assessment tool	Follow-up
Singh et al. (2025) [17]	Split-mouth RCT	January 2019 to December 2022	Patients requiring bilateral MTM extraction.	Adults aged <45 years with bilateral impacted MTMs (Pederson’s score ≤ 6)	Substance abuse, local anesthetic allergy, and uncontrolled medical conditions (ASA ≥ II)	Magnesium sulphate (MgSO_4_): 1.8 mL 2% lignocaine + 0.2 mL 50% MgSO_4_ for IANB	Placebo	Adjuvant	VAS	24 and 72 hours postoperatively, and on day 7
Jerkovic et al. (2020) [14]	Split-mouth RCT	April 2017 to January 2019	Patients requiring bilateral MTM extraction.	Healthy adults aged >18 years with bilateral impacted MTMs of similar surgical difficulty	Pregnancy, breastfeeding, drug abuse, and use of other medications/supplements	Oral: two arms: (1) 400 mg magnesium citrate tablets daily for 3 days. (2) 100 mg magnesium citrate lozenges every 6 hours for 3 days	Placebo	Oral	VAS	24 hours, 48 hours, and 72 hours postoperatively
Nimkulrat et al. (2025) [15]	Split-mouth RCT	NA	Patients requiring bilateral MTM extraction.	Healthy adults aged >18 years with bilateral MTMs requiring bone removal and tooth sectioning	Pregnancy, breastfeeding, systemic diseases contraindicating magnesium, and ongoing orofacial pain	Oral: 500 mg magnesium supplement (oxide, citrate, succinate) once daily for 3 days as an adjuvant to ibuprofen	Placebo	Adjuvant	HP-VAS	6 hours, 24 hours, 48 hours, and 72 hours postoperatively
Powcharoen et al. (2025) [16]	Parallel-group RCT	December 2021 to April 2023	Patients undergoing single MTM extraction.	Healthy adults aged >18 years with an impacted MTM.	Pregnancy, lactation, contraindications to study drugs, and ongoing orofacial pain	Magnesium sulphate (MgSO_4_): IANB with 4% articaine plus either 150 mg or 250 mg of MgSO_4_	Placebo	Adjuvant	HP-VAS	6 hours and 24 hours postoperatively
Tejashree et al. (2021) [18]	Parallel-Group RCT	May 2019 to April 2020	70 patients undergoing single MTM surgery. Mean age ~28 years	Healthy adults (18–30 years, ASA I) with an impacted MTM	Long-term medication use, recent steroid/antibiotic use, pregnancy, lactation, anticoagulant therapy	Oral: 100 mg magnesium lozenge 30 minutes preoperatively and every 6 hours for 72 hours	Placebo	Oral	VAS	6 hours, 24 hours, 48 hours, and 72 hours postoperatively

**Figure 2:**
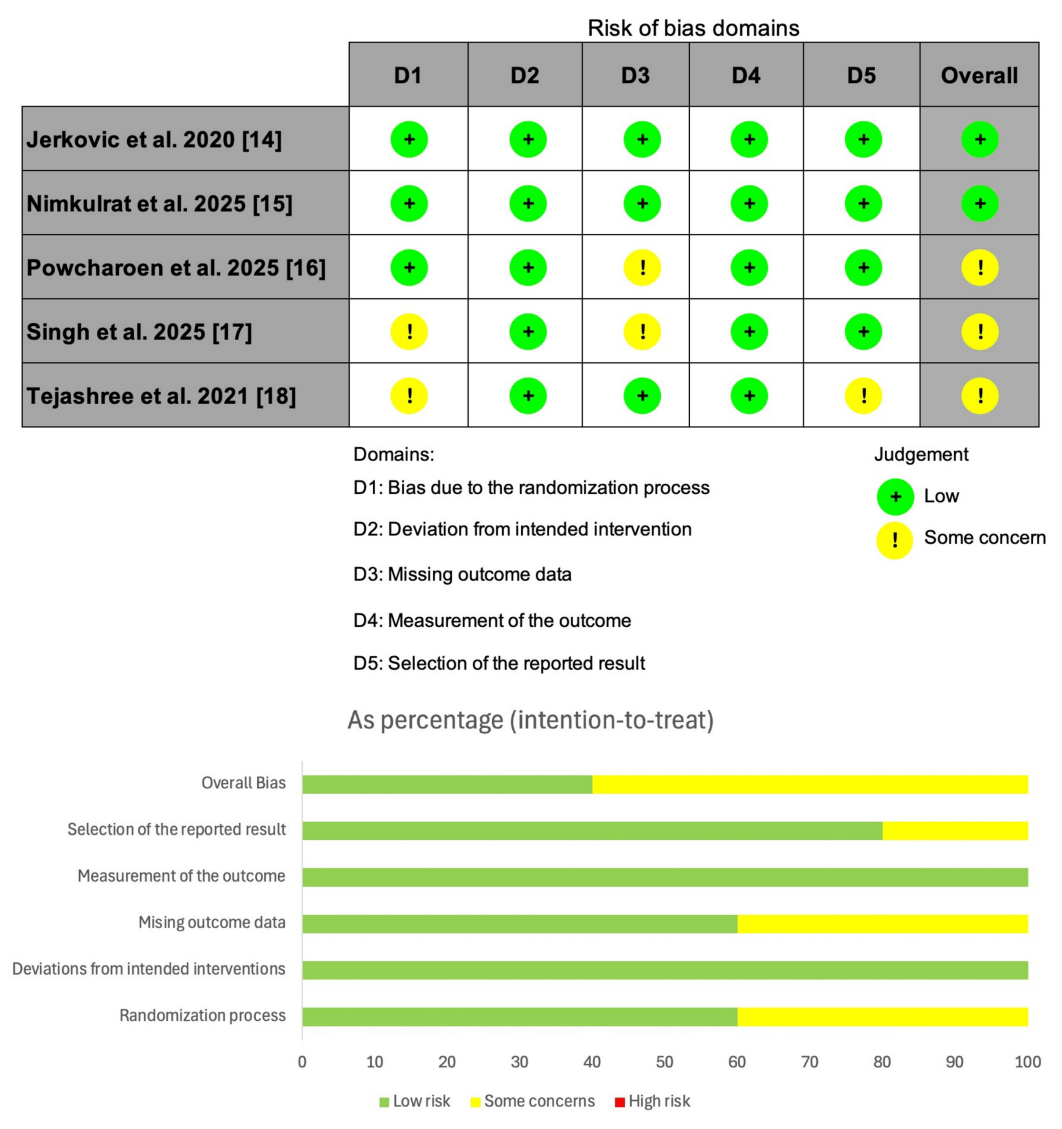
Summary of the risk of bias of the included studies. Cochrane risk of bias tool version 2 for randomized trials.

All included RCTs were assessed using the ROB-2 tool, with only two trials having a low risk of bias. Meanwhile, the other three trials had some concerns regarding the randomization process, missing outcome data, and selection of the reported results (Figure 2).

Outcomes

**Figure 3:**
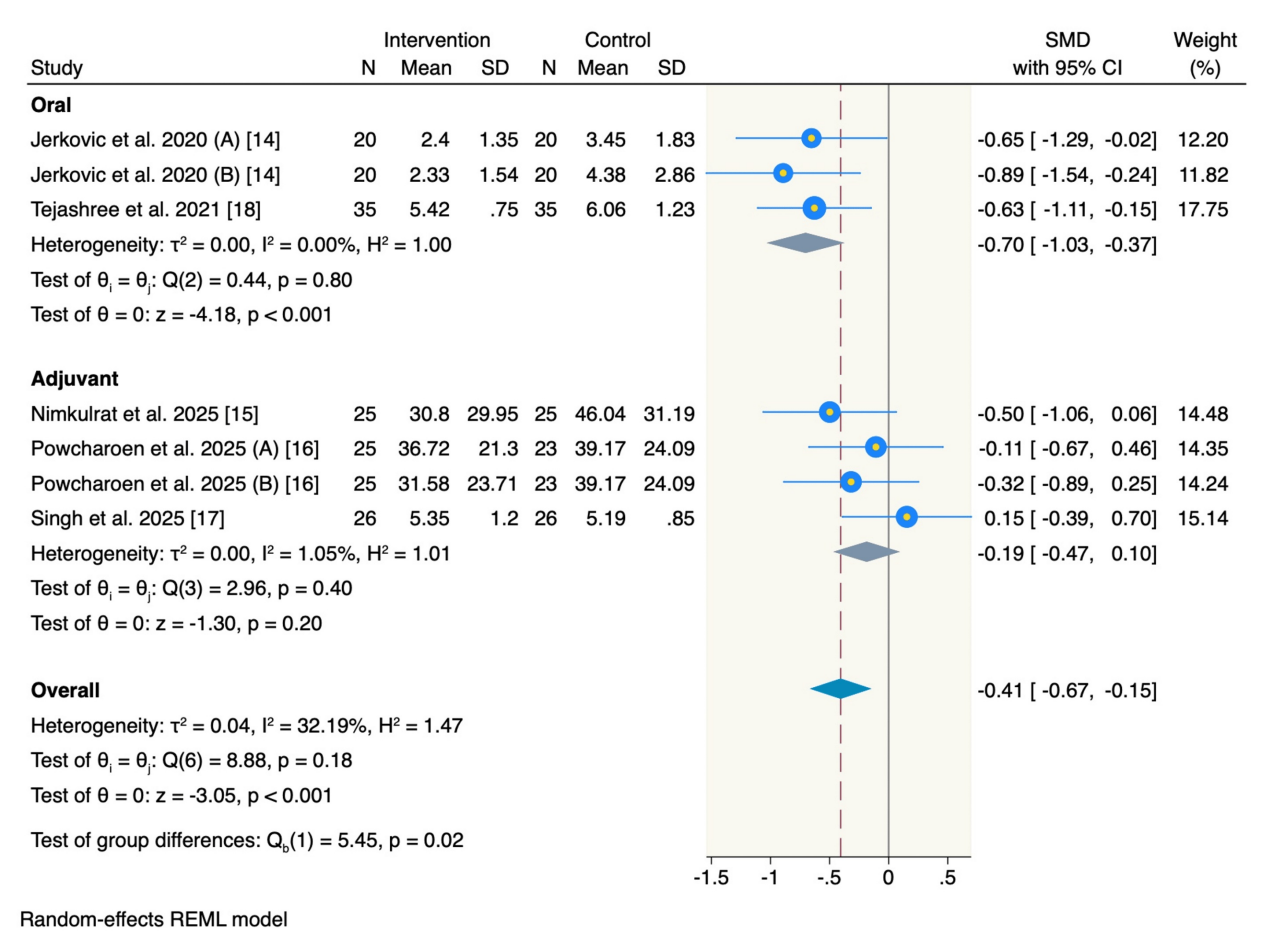
Forest plot of postoperative pain scores at 24 hours.

Postoperative pain score: All five RCTs assessed the pain score at 24 hours post-operation. Subgroup analysis showed that oral magnesium was associated with a significant reduction in pain scores compared to the control group (SMD = -0.7, 95% CI = -1.03 to -0.37, p < 0.001; I² = 0%), meanwhile, no significant difference between the adjuvant magnesium and control group was observed (SMD = -0.19, 95% CI = -0.47 to 0.1, p = 0.2; I² = 1.05%). The overall pooled estimate showed a significant decrease in the pain score at 24 hours in the overall intervention group (SMD = -0.41, 95% CI = -0.67 to -0.15, p < 0.001; I² = 32.19%) (Figure 3).

**Figure 4:**
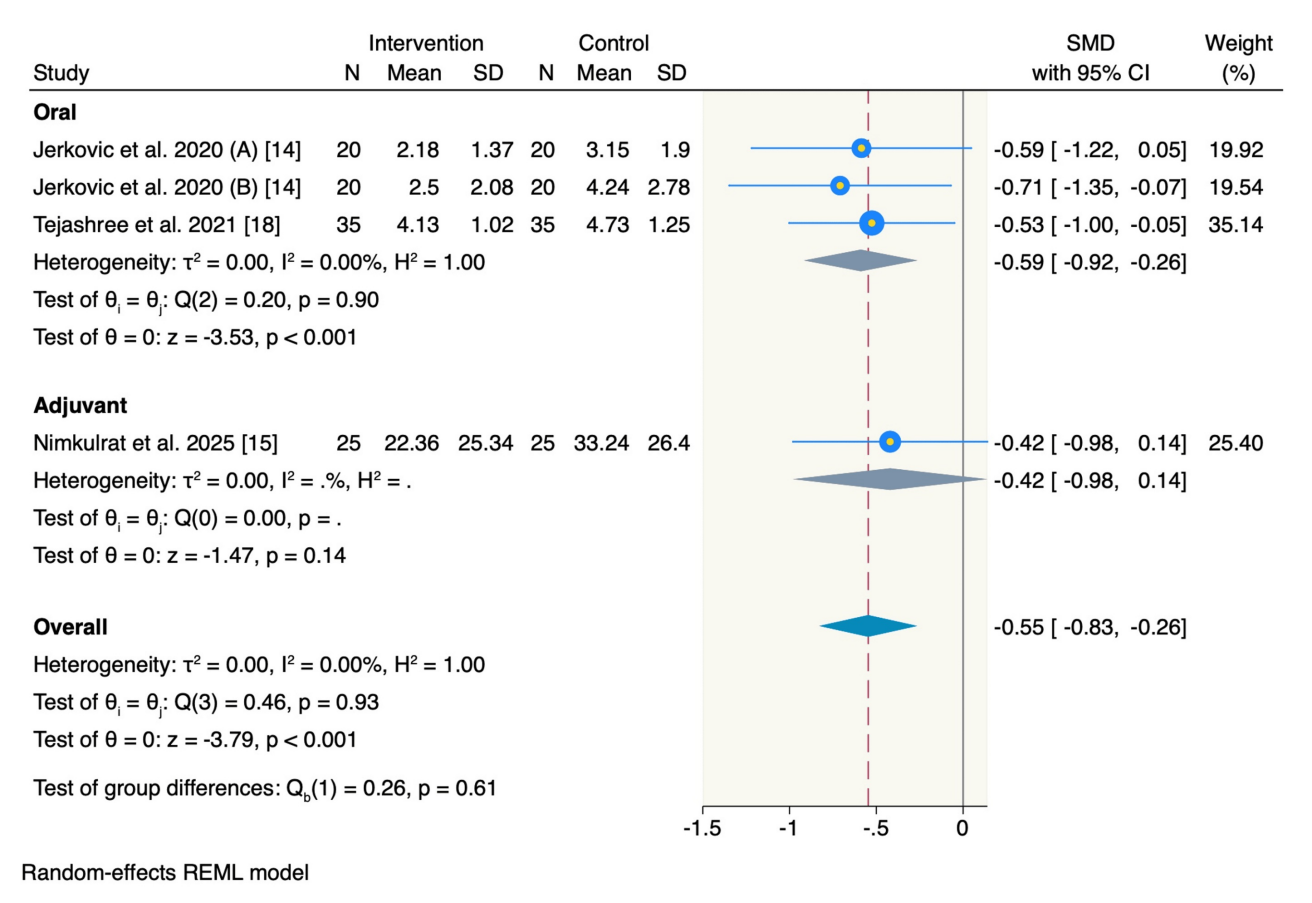
Forest plot of postoperative pain scores at 48 hours.

Only three studies reported the pain score at 48 hours post-operation, with a subgroup analysis showing a significant decrease in pain scores following the administration of oral magnesium compared to the control group (SMD = -0.59, 95% CI = -0.92 to -0.26, p < 0.001; I² = 0%). However, it showed no significant difference between the adjuvant use of magnesium and the control group (SMD = -0.42, 95% CI = -0.98 to 0.14, p = 0.14; I² = 0.00). The overall pooled estimate showed a significant decrease in the pain scores in the overall intervention group compared to the control group (SMD = -0.55, 95% CI = -0.83 to -0.26, p < 0.001; I² = 0%) (Figure 4).

**Figure 5:**
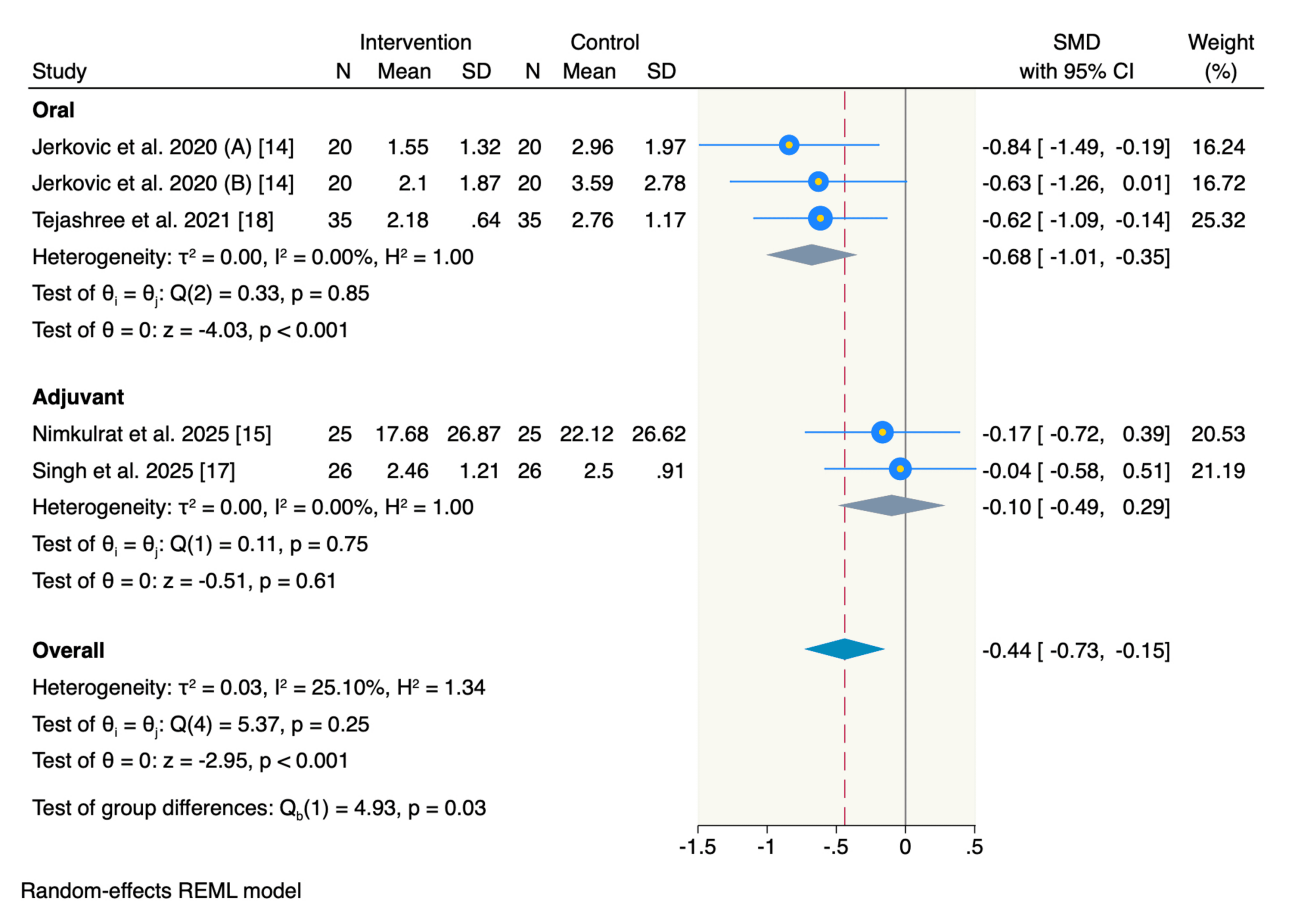
Forest plot of postoperative pain scores at 72 hours.

Four studies reported pain scores at 72 hours post-operation, with a subgroup analysis showing a significant reduction in pain scores following oral magnesium compared to the control group (SMD = -0.68, 95% CI = -1.01 to -0.35, p < 0.001; I² = 0%). Meanwhile, it showed no significant difference between the adjuvant use of magnesium and the control group (SMD = -0.1, 95% CI = -0.49 to 0.29, p = 0.61; I² = 0%). However, the overall pooled estimate showed a significant decrease in the pain scores at 72 hours post-operation among the overall intervention group patients compared to the control group (SMD = -0.44, 95% CI = -0.73 to -0.15, p < 0.001; I² = 25.1%) (Figure 5).

**Figure 6:**
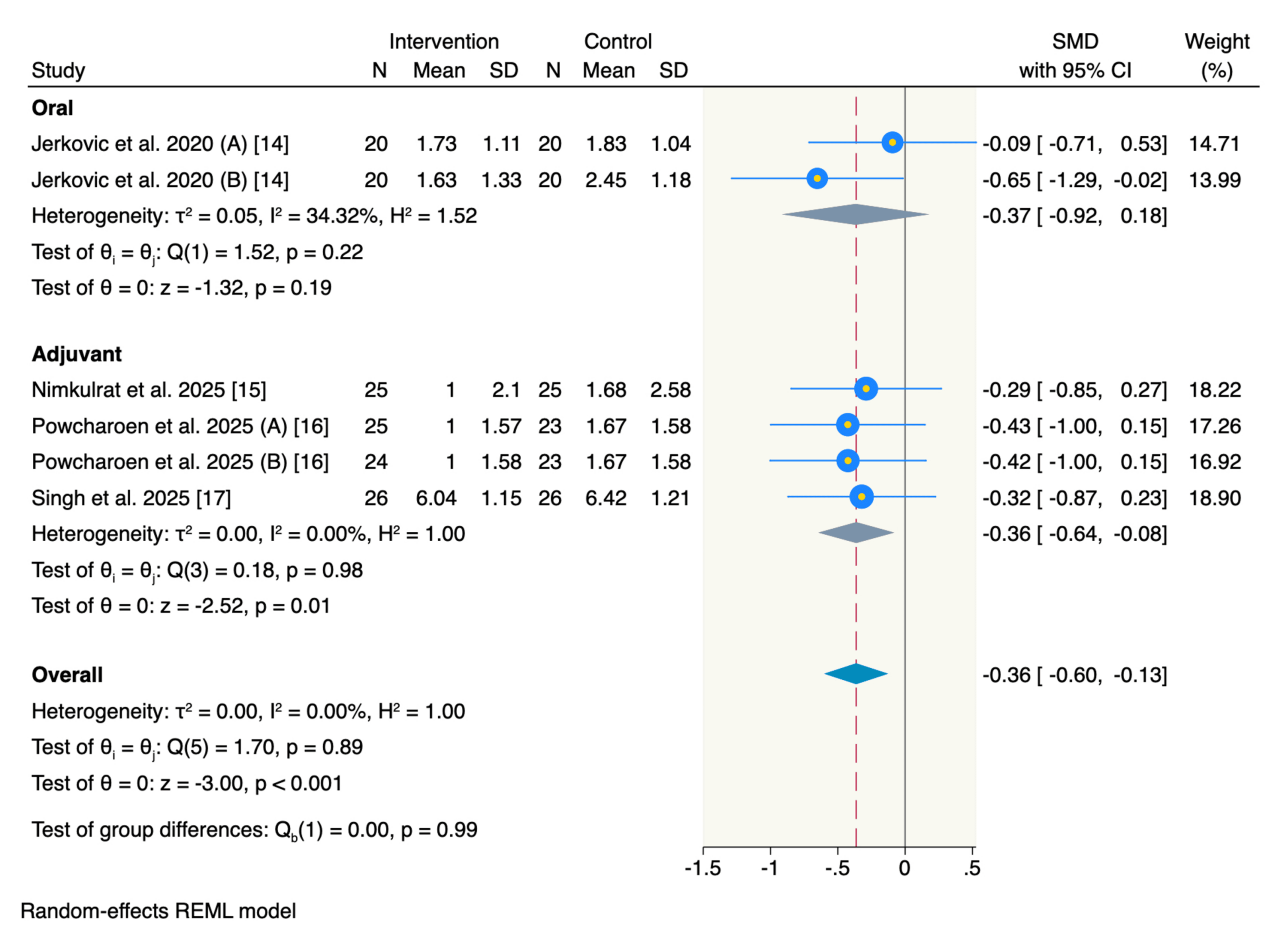
Forest plot of number of consumed analgesics postoperatively.

Number of consumed analgesics: Four RCTs reported the mean number of consumed analgesics, with a subgroup analysis showing no significant difference between oral use of magnesium and the control group (SMD = -0.37, 95% CI = -0.92 to 0.18, p = 0.19; I² = 34.32%). However, it showed a significant decrease in analgesic consumption following the adjuvant use of magnesium compared to the control group (SMD = -0.36, 95% CI = -0.64 to -0.08, p = 0.01; I² = 0%). The overall pooled estimate showed that patients in the intervention group needed significantly fewer analgesics compared to the control group (SMD = -0.36, 95% CI = -0.6 to -0.13, p < 0.001; I² = 0%) (Figure 6).

**Figure 7:**
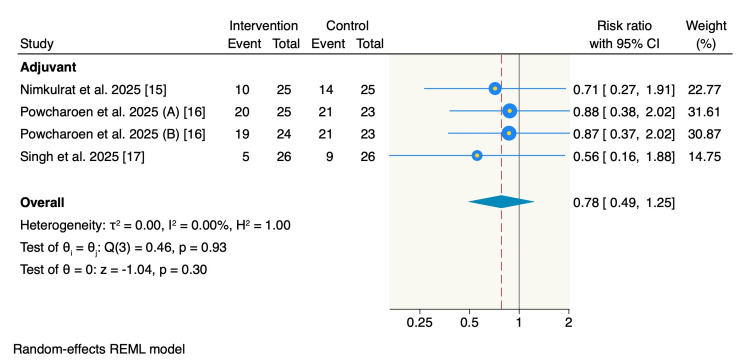
Forest plot of the number of the patients who received supplemental analgesics postoperatively.

Number of patients receiving analgesics: Only three RCTs reported the number of patients who required supplemental analgesics, with an event rate of 54 out of 100 (54%) patients in the intervention group and 44 out of 74 (59.5%) patients in the control group. The pooled estimate showed no significant difference between the adjuvant use of magnesium and the control group (RR = 0.78, 95% CI = 0.49 to 1.25, p = 0.3; I² = 0%) (Figure 7).

Discussion

Our systematic review and meta-analysis of 299 patients is the first and the most comprehensive study to compare the oral and adjuvant use of magnesium to IANB in patients undergoing lower third molar surgery. Our pooled analysis showed that the oral use of magnesium was associated with lower pain scores at 24 hours, 48 hours, and 72 hours following the procedure compared to the control group. Additionally, the adjuvant use of magnesium was associated with a lower number of consumed analgesics compared to the control group. On the other hand, no significant differences were observed in other reported outcomes.

Patients undergoing molar surgeries often experience severe pain during and after the procedure. Moreover, this pain represents a complex phenomenon that often requires a complex assessment to attenuate the pain scores in these patients. During surgical procedures, the process of pain transmission from the molar area to the central nervous system is mediated by voltage-gated sodium channels [19] and is regulated by numerous substances, such as bradykinin and prostaglandin E2. By blocking these channels or controlling the release of these substances, there is a higher chance of IANB success during these surgical procedures [7]. Magnesium is a key modulator that is currently being used in routine clinical practice either in the oral form or as an adjuvant solution to IANB, due to its higher effect to increase the analgesic efficacy and the success rates of IANB [20]. The main advantages of magnesium are the antagonizing effect of NMDA receptors and the significant reduction of catecholamine release [20].

We reported a significant reduction in pain scores following the administration of oral magnesium compared to the control group at different follow-up durations, without the beneficial effect of the adjuvant use of magnesium to reduce the pain scores, highlighting the superior effect of the oral form of magnesium over the adjuvant form. However, the adjuvant use of magnesium was associated with lower rates of supplemental analgesia use compared to the control group, without a difference regarding the oral form. The notable difference could be explained by the local effectiveness of the oral form. The oral form of magnesium, which is a common form of magnesium citrate, is converted into a highly soluble form, making magnesium very easily and readily ionized, which is the most active form of magnesium to be dissolved into tissues [21]. Borazan et al. reported that in the case of direct contact of the ionized form of magnesium with the pharyngeal wall, the effect of magnesium might begin immediately and continue to be absorbed from the pharyngeal mucosa until it is in the tissue [21]. Following this process, magnesium acts as a local analgesic with a significant anti-inflammatory effect, which is exacerbated in the form of inflammation or a high degree of pain, especially during molar surgeries [22]. Additionally, due to the similarity in the structure of oral mucosa and the pharyngal mucosa, a similar magnesium effect could be applied to the surgical procedures of the oral cavity [23].

Moreover, the bioavailability of magnesium plays a key role in the analgesic efficacy. It depends mainly on the form of magnesium being administered. The bioavailability of the organic oral magnesium mixtures as magnesium citrate is notably better than inorganic forms of magnesium being administered as an adjuvant form, with the efficiency of magnesium being better when highly ionized forms are used [24]. Additionally, the ingestion of multiple low doses of magnesium over the course of the day leads to greater relative bioavailability compared to single high-dose ingestion [25]. This might explain our findings that magnesium significantly reduced pain scores at 24 hours following the procedure, not earlier.

Our findings are aligned with the previous studies of Jerkovic et al. [14] and Nimkulrat et al. [15]; however, some differences were noted. Jerkovic et al. [14] reported that the analgesic efficacy of oral magnesium once daily for three days significantly reduced the pain scores across the entire three-day follow-up, with additional reduction of rescue analgesic consumption. However, Nimkulrat et al. [15] reported only a significant reduction in pain scores on the first day of assessment post-surgery, and failed to decrease the rescue analgesic consumption. The difference might be attributed to the different composition of the pharmaceutical forms of administered magnesium between the two studies. Moreover, the results of Nimkulrat et al. [15] should be interpreted with caution, as the reported upper CI of the effect estimate of pain reduction was below the expected minimal clinically important difference, which could be translated into a non-clinical significant result [26].

On the other hand, the administration of magnesium orally can be associated with notable adverse events such as diarrhea or general gastrointestinal adverse reactions [27], which was previously documented at high magnesium doses [27]. Therefore, the US Food and Drug Administration recommends a daily dose of magnesium of 420 mg for adults and children aged more than four years [28]. Most included studies followed the same recommendation; however, Nimkulrat et al. [15] found no adverse events following the administration of magnesium.

In addition, the increasing use of combining non-steroidal anti-inflammatory drugs with oral magnesium was tested across the included studies, reporting a significant control over undesirable pain, especially during the first postoperative day; however, from a clinical perspective, these results should be interpreted carefully.

Limitations

Although the present systematic review and meta-analysis is the most comprehensive study to outline the effectiveness of the use of magnesium to reduce postoperative pain scores compared to the control group, some limitations should be addressed before generalizing our findings. First, the included studies used different doses of magnesium with different pharmacological forms as oral or lozenge, which could raise potential differences in the reported effect estimates. Despite no notable effect of the dose on our pooled estimates, the standardized approach should be generalized with more consistent findings over the follow-up duration. Second, the sample size of the included studies was relatively small, indicating the need for larger studies to further consolidate the benefits of magnesium in this setting. Despite consistent findings up to 72 hours, more studies with longer follow-up durations are needed to consolidate the findings over a longer time period. While the studies used standardized protocols, some variations in the clinical setting were noticed, especially the initial pain severity of the included patients, which could be a source of heterogeneity.

## Conclusions

The present systematic review and meta-analysis showed that the administration of oral magnesium was associated with significantly lower pain scores at 24 hours, 48 hours, and 72 hours post-procedure, without significant differences regarding the adjuvant form of magnesium. Additionally, the adjuvant magnesium form was associated with lower analgesic consumption compared to the control group. Further RCTs with standardized protocols are needed to validate the current findings.
